# Temporal changes in the vaginal microbiota in self-samples and its association with persistent HPV16 infection and CIN2+

**DOI:** 10.1186/s12985-020-01420-z

**Published:** 2020-10-07

**Authors:** Malin Berggrund, Inger Gustavsson, Riina Aarnio, Julia Hedlund Lindberg, Karin Sanner, Ingrid Wikström, Stefan Enroth, Ignas Bunikis, Matts Olovsson, Ulf Gyllensten

**Affiliations:** 1 Department of Immunology, Genetics, and Pathology, Biomedical Center, Science for Life Laboratory (SciLifeLab) Uppsala, Uppsala University, Box 815, 75108 Uppsala, Sweden; 2 Department of Women’s and Children’s Health, Uppsala University, 75185 Uppsala, Sweden; 3 Uppsala Genome Center, Science for Life Laboratory, Department of Immunology, Genetics, and Pathology, Uppsala University, BMC, Box 815, 752 37 Uppsala, Sweden

**Keywords:** Vaginal microbiota, Self-sampling, HPV, CIN2+, Cervical cancer, FTA card

## Abstract

**Background:**

The vaginal microbiota has been reported to be associated with HPV infection and cervical cancer. This study was performed to compare the vaginal microbiota at two timepoints in women performing self-sampling and had a persistent or transient HPV16 infection. The women were tested for 12 high-risk HPV (hrHPV) types but only women with single type (HPV16) were included to reduce confounding variables.

**Methods:**

In total 96 women were included in this study. Of these, 26 were single positive for HPV16 in the baseline test and HPV negative in the follow-up test and 38 were single positive for HPV16 in both tests and diagnosed with CIN2+ in histology. In addition, 32 women that were negative for all 12 HPV tested were included. The samples of vaginal fluid were analyzed with the Ion 16S™ Metagenomics Kit and Ion 16S™ metagenomics module within the Ion Reporter™ software.

**Results:**

K-means clustering resulted in two *Lactobacillus*-dominated groups, one with *Lactobacillus sp.* and the other specifically with *Lactobacillus iners*. The two remaining clusters were dominated by a mixed non-*Lactobacillus* microbiota*.* HPV negative women had lower prevalence (28%) of the non-Lactobacill dominant cluster in the baseline test, as compared to women with HPV16 infection (42%) (*p* value = 0.0173). Transition between clusters were more frequent in women with persistent HPV16 infection (34%) as compared in women who cleared the HPV16 infection (19%) (*p* value = 0.036).

**Conclusions:**

The vaginal microbiota showed a higher rate of transitioning between bacterial profiles in women with persistent HPV16 infection as compared to women with transient infection. This indicate an instability in the microenvironment in women with persistent HPV infection and development of CIN2+.

## Introduction

Persistent infection of human papilloma virus (HPV) is the main cause for development of cervical cancer [1]. A set of HPV types have oncogenic properties and are considered high-risk HPV [2], with HPV16 being the most prevalent type in most regions [3].

In addition to HPV, other risk factors for cervical cancer is recognized, such as smoking, high parity and use of oral contraceptives [4]. The association between HPV infection and the vaginal microbiota is not fully understood. Using 16S sequencing, the vaginal microbiota profiles has previously been described as five community state types (CST). CST I, II, III and V are dominated by *Lactobacillus crispatus*, *L. gasseri*, *L. iners* and *L. jensenii*, respectively, while CST IV is dominated by non-*Lactobacillus* bacteria [5]. CST IV is further subgrouped into CST IV-a and IV-b, with IV-a having modest proportions of *Lactobacillus *spp*.* and low proportions of strictly anaerobic bacteria, while IV-b having higher proportions of *Atopobium,* as well as *Prevotella, Sneathia* and *Gardnerella* [6]*.*

Several studies report *Atopobium vaginae* and *Gardnerella vaginalis* to be associated with cervical interepithelial neoplasia (CIN) [7–9]. However, other results show that decline of *Gardnerella* abundance with HPV positivity and increasing grade of CIN is associated [10]. Lower abundance of *Lactobacillus sp.* and higher frequency of CST-IV is associated with HPV infection and grade of CIN in several studies [11–13]. CST II (*L. gasseri*-dominated) is related to fast HPV clearance rate, and CST IV-B with slow HPV clearance rate, in comparison to CST I [14]. Also, one study reports relative abundance of *L. Crispatus* to be associated with lower HPV detection rate, but finds no association between HPV and relative abundance of other *Lactobacillus* species or with *Lactobacillus* as a group [15].

The vaginal microbiota in women is known to also depend on covariates such as the menstrual cycle [6, 16], pregnancy [17], use of hormonal contraceptives [18], sexual behavior and smoking [19]. In addition, the results can be affected by technical variations such as choice of variable regions of the 16S gene [20] and sequencing platform [21].

The present study was based on analyses of seven variable domains of the 16S RNA gene in single samples from women with HPV negative tests and paired vaginal samples collected 4–6 months apart from women with a persistent HPV16 infection and women with transient infections. Only women infected by a single hrHPV type (HPV16) were included to reduce confounding variables in the analysis. These women were also tested for HPV18, 31, 33, 35, 39, 45, 51, 52, 56, 58 and 59 with negative results. The aim was to study the association of the vaginal microbiota with the course of HPV16 infection.

## Methods

### Study population and samples

Women in this study were selected from a previous randomized intervention study conducted between 2013 and 2015, that included non-pregnant women between 30 and 49 years of age from Uppsala County, Sweden [22]. To be eligible for the randomized study, the woman had to be between 30 and 49 years at entry (date of invitation), having no previous hysterectomy, no current pregnancy and no clinical test results (Pap smear cytology, HPV test or histology) relating to cervical cancer registered within 1 year before the date of invitation.

In brief, the randomized intervention study consisted of a total of 36,390 women out of which 17,997 were randomized to repeated HPV-testing with self-sampling. 7997 women performed the 1st test with 7443 negative and 554 positive tests. The aim of the previous study was to compare the detection rate of CIN2+ in histology in women performing repeated self-sampling of vaginal fluid for HPV testing, with the rate of CIN2+ detection in women following the regular screening program based on Pap smear cytology. Women who were HPV positive (N = 554) in their first test were asked to repeat the self-sampling in 4–6 months. 501 of the 554 women followed the study protocol and performed a 2nd HPV-test which resulted in 355 positive tests. Women that were HPV negative in the first (N = 7443) or second HPV test (N = 146) were referred to the regular screening program. For the present study we selected 26 women who were single infected by HPV16 in the baseline test and subsequently HPV negative in their follow-up test, and 38 women who were single infected by HPV16 in both the baseline and the follow-up tests, and later diagnosed as CIN2+ based on histology during the follow-up period of 18 months from invitation date. Finally, 32 age-matched women who had a single self-sample with a negative HPV test were also included. Women that were infected by HPV16 in their baseline test and HPV negative in their follow-up test were considered to have transient infections, while women with two single HPV16 positive samples together with CIN2+ histology were considered to have a persistent infection. The study was approved by the Regional Ethics Committee in Uppsala (Dnr 2012/099).

### Sample collection and processing

The vaginal self-sampling procedure has been described earlier [23]. Briefly, the women performed sample collection using the Rovers®Viba-brush (Rover Medical Devices B.V., Oss, The Netherlands) and applied the sample to the indicating FTA elute micro card™ (art. no WB129308, GE Healthcare, Longwood Dr, Cardiff CF14 7YT, UK). The instruction was to (1) insert the Rovers Viba‐brush approximately 5–10 cm into the vagina and gently turn it one full circle, (2) remove the brush and apply the vaginal sample to the FTA elute micro card ™ by placing the brush in the middle of the application area and rolling it one full circle across that area and, (3) air‐dry the FTA card for a few minutes, fold the lid, put it in the enclosed envelope and send it to the HPV laboratory by regular mail. All samples were analyzed at the Department of Immunology, Genetics and Pathology, Uppsala University, Sweden. The FTA cards were processed using an automated laboratory system (easyPunch STARlet, Hamilton Robotics, Via Crusch 8 CH‐7402 Bonaduz, GR, Switzerland), where a robot arm picks up each card, takes a photograph of the sampling area and then identifies which parts of the card that contains the highest concentration of cellular material, using a machine learning software. The robot then positions the card in a punching device and collects four circular pieces of 3 mm diameter in a single well in a 96‐well microtiter plate. DNA was extracted from the card punches as previously described [24, 25].

### HPV DNA typing

A clinically validated qPCR‐based assay, HPVIR [26], was used for HPV DNA testing. The test detects and quantifies the following HPV types: 16, 18, 31, 33, 35, 39, 45, 51, 52, 56, 58 and 59. The limit of detection (LOD) for HPV is 10 HPV copies per PCR. This test also detects and quantifies a human single copy gene (housekeeping gene), HMBS (*Homo sapiens* hydroxymethylbilane synthase; GenBank accession no. M95623.1) as a control for the amount of human cellular material. The LOD for human genomic DNA is 10 copies of HMBS per PCR.

### Colposcopy and histology

The colposcopic evaluation included an identification of squamocolumnar junction and transformation zone (TZ) with application of 5% acetic acid and iodine solution. Biopsies were obtained from all the identified abnormal areas, and a blind biopsy was taken in women with normal colposcopy. All gynecological examinations were performed at the Clinic of Obstetrics and Gynecology, Uppsala University Hospital. Clinical classifications were according to SNOMED (Systematized, Nomenclature of Medicine; College of American Pathologists, Skokie, IL, USA) and the highest histological grade found in each patient was used for interpretation of the results.

### Sample preparation and 16S rRNA gene Ion Torrent amplicon sequencing

16S hypervariable regions were amplified using Ion 16S™ Metagenomics Kit (Thermo Fisher Scientific) following recommendations in the *Ion 16S™ Metagenomics Kit User Guide* revision C.0. According to the manufacturer’s instructions, 3.0 μl of the FTA-card eluate was used as input to the amplification. After purification with Agencourt® AMPure® XP beads, amplicons were quantified using Bioanalyzer instrument (Agilent) and DNA-concentrations in the products ranged from 1.6 to 77.3 ng/μl. With the exception of one sample where 50 ng was used, 100 ng of the input material was used for the AB Library Builder™ System (Thermo Fisher Scientific), following the guide *Ion Xpress™ Plus and Ion Plus Library Preparation for the AB Library Builder™ System* revision 5.0. Prepared libraries were amplified for addition 5 cycles as described in *Ion 16S™ Metagenomics Kit User Guide* revision C.0. Final libraries were quantified using the Fragment analyzer (Agilent) and prepared in the Ion Chef System (Thermo Fisher Scientific) before sequenced on the Ion S5 ™ XL System (Thermo Fisher Scientific) on five Ion 530™ Chips (Thermo Fisher Scientific).

The primary data analysis was carried out with The Ion reporter version 5.6. Briefly, quality analysis was run requiring primer detection in both ends, the minimum alignment coverage of 90%. The analysis was performed with the built in QIIME pipeline with protocol Metagenomics 16S w1.1. Reference library Curated Greengenes v13.5 was used and a minimum alignment of 97% was required for genus identification and 99% for species identification. If no identification at 99% could be made on species level but was achieved at genus level, genus level annotations were used. Difference between the two top hits was allowed to be maximum 0.2% to confirm species identification.

### Statistical analysis

Statistical calculations were performed and figures generated using R version 3.4.3 [27]. Read number for each operational taxonomic unit (OTU) was normalized for total number of reads for each sample, e.g. reported in proportions of total number of reads. Non-*Lactobacillus* bacteria were grouped by taxonomic level genus if information was available, otherwise they were grouped on taxonomic level family. Bacterial genus or family that represented less than 1% of the total were grouped as “Others” for non-*Lactobacillus* bacteria, and as “Lactobacillus sp.2” for *Lactobacillus* bacteria. K-means clustering were performed with four clusters, using the “kmeans”-function and the results visualized as a heatmap using the “pheatmap”-package [28]. Shannon index for alpha diversity was calculated with the “vegan”-package [29]. Sankey plots were visualized with the package “rCharts” [30]. Binomial testing was used for comparing cluster distribution between sample groups. *p* values were adjusted for multiple testing using the Bonferroni correction and *q* values were considered significant if the *q* value < 0.05. Binomial testing was used for comparison of cluster transition proportions.

## Results

### Sample characteristics

The median time between the two self-sampling occasions were 162 days and the mean absolute deviation (mad) 14.8 days for women with transient HPV16 infection and 155.5 days (mad = 30.4) for women with persistent HPV16 infection. The number of days between the two sample collections was not significantly different between women with transient and persistent infection (*p* value = 0.35). Also, the mean age was not significantly different between HPV negative women (mean = 36.7, SD = 5.2), women with transient HPV16 infection (mean = 35.9, SD = 5.2) and women with persistent HPV16 infection and CIN2+ (mean = 37.5, SD = 6.0, *p* value = 0.31–0.64) (Table 1).

**Table 1:** Basic characteristics of samples from HPV negative women, women with transient HPV16 infection and women with persistent HPV16 infection and CIN2+

	HPV negative	Transient HPV16 infection	Persistent HPV16 infection
Number of samples	32	26	38
Mean age^a^ (SD)	36.7 (5.2)	35.9 (5.2)	37.5 (6.0)
Sampling distance^b^ (SD)	NA	184 (79)	165 (33)
Comparisons			
HPV16 positive vs. HPV negative (*p* values)			
Age^c^		0.525	0.64
Persistent vs. transient (*p* values)			
Age^c^			0.309
Sampling distance^c^			0.352

^a^Age in years, given as: mean (standard deviation, SD)

^b^Distance between base-line sample and follow-up sample in days, given as: mean (standard deviation, SD)

^c^Two-sided Wilcoxon’s test

### 16S rRNA gene sequencing and comparison between 16S regions V2-V9 and V3/V4

The Ion 16S™ Metagenomics Kit targets seven variable regions (V2, V3, V4, V6, V7, V8 and V9). The number of reads per sample across these seven regions was between 125,202 and 1,148,323, with a mean of 515,814 ± 190,890 reads. Based on the analysis of all seven variable regions, the analysis identified 723 operational taxonomic units (OTUs), corresponding to 433 species, 188 genera and 102 families. When the analysis was based solely on regions V3 and V4, which is a set of 16S variable regions frequently used for analysis, we identified 547 OTUs, representing 324 species, 157 genera and 66 families (Additional file 1: Figure S1a–c). For regions V3 and V4 the number of reads per sample was between 68,211 and 574,180, with a mean of 277,874 ± 103,597. Thus, including the seven regions resulted in the identification of additional OTUs at all three taxonomic levels. For instance, using the seven regions resulted in the identification of 34 *Lactobacillus* species, whereas using only regions V3 and V4 resulted in 26 *Lactobacillus* species. Since the analysis based on seven regions identified a higher number of OTUs at all three taxonomic levels and, in particular, a higher number of *Lactobacillus* species, we based the subsequent analysis on all seven 16S regions. Using all seven region the number of OTUs per sample varied between 5 and 215, with a mean of 53 OTUs per sample.

### K-means clustering and cluster characteristics

**Fig. 1:**
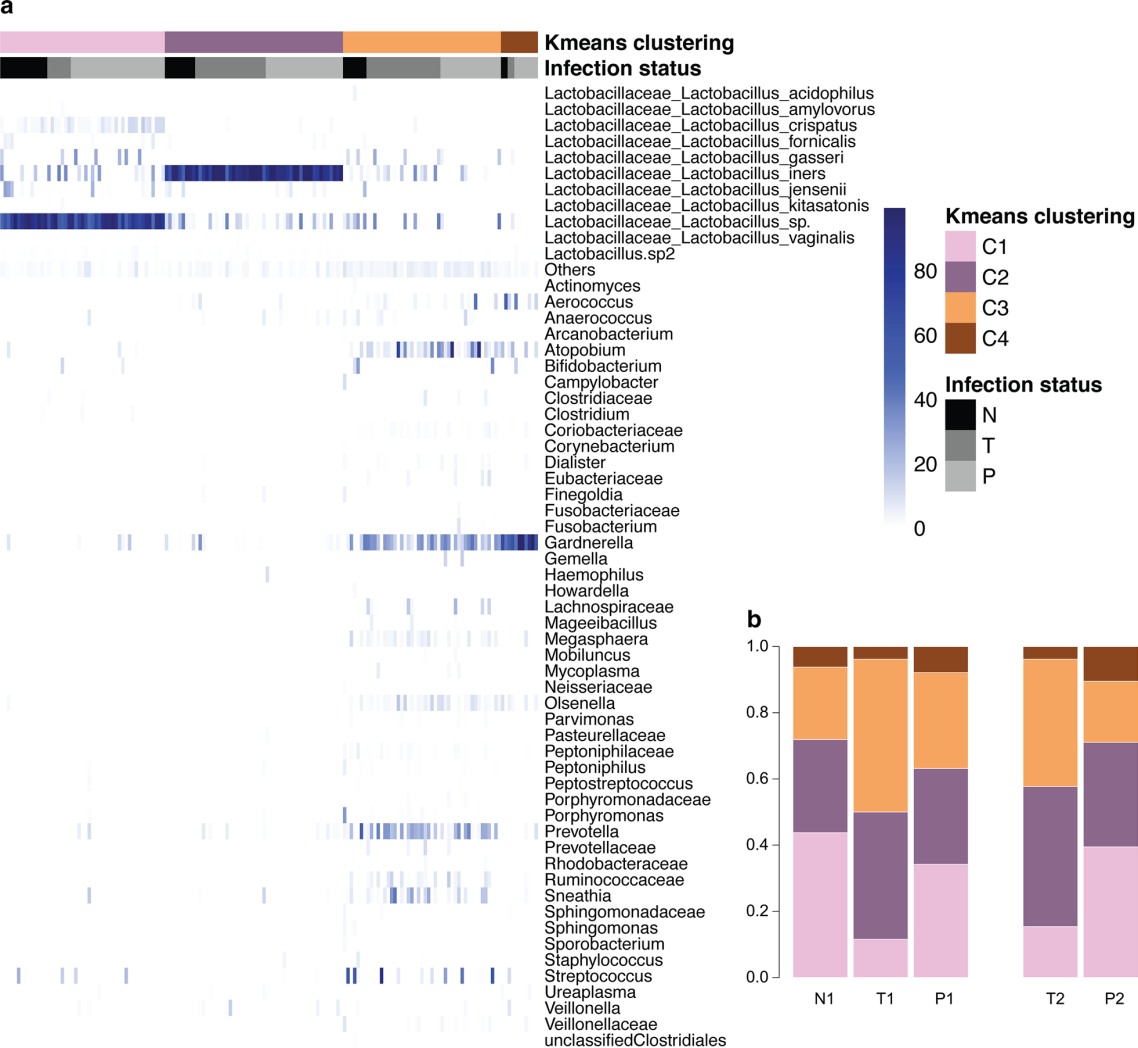
**a** Heatmap of relative abundance of bacterial taxa. Each column represents a sample and each row a bacterial taxon. The top box indicates K means clustering belonging (C1–C4) and the second box from the top indicates infection group belonging (N = HPV negative, T = Transient HPV16 infection, P = Persistent HPV16 infection). *Lactobacillus *sp. are reported at highest possible taxonomic level and other bacteria are grouped at genus level if known, otherwise they are grouped on family level. Bacteria with lower abundance than 1% are classified as “Others” if they are non-*Lactobacillus*, and “Lactobacillus.sp2.” if they belong to *Lactobacillus* sp. **b** Stacked barplot for each infection group and sampling round (N = HPV negative, T = Transient HPV16 infection, P = Persistent HPV16 infection, 1 = Baseline sample, 2 = Follow up sample. Color indicate K means clustering group (C1–C4)

All OTUs with an abundance below 1% within a sample were analyzed as a group. OTUs belonging to *Lactobacillus sp.* and non-*Lactobacillus *sp. were grouped separately. K-means clustering, using all samples, with four clusters resulted in two *Lactobacillus*-dominated clusters. In the first cluster, samples had OTUs with unclassified *Lactobacillus *sp*.* as the most dominant species, with a mean proportion of 76.6% (min = 45.9%, max = 99.3%), and in the second cluster samples were dominated by OTUs specifically classified as *Lactobacillus Iners,* with a mean proportion of 86.4% (min = 48.2%, max = 99.4%). In cluster 3 and 4, samples were dominated by a mixed non-*Lactobacillus* microbiota. In the third cluster most samples were dominated by *Gardnerella* or *Prevotella* and some samples were dominated by e.g. *Atopobium*, *Snethia* or *Streptococcus*, with a mean proportion for the dominant bacteria of 39.4% (min = 15.2%, max = 92.9%). Finally, in the fourth cluster ten of eleven samples were dominated by *Gardnerella* and the last sample by *Aerococcus,* with a mean proportion of the dominant bacteria of 69.8% (min = 43.0%, max = 99.4%) (Fig. 1a).

### Vaginal microbiome clusters in HPV negative women and those with transient or persistent HPV16 infection

In the baseline test, HPV negative women had a lower prevalence (28%) of the non-Lactobacilli dominant cluster (cluster 3 and 4), as compared to women with an HPV16 infection (42%). This difference was nominally statistically significant (*p* value = 0.0173), but the difference did not remain significant when corrected for multiple testing (Bonferroni normalized *q* value = 0.0519) (Table 2). In the second sample, women with transient infection had higher prevalence (42%) of the non-*Lactobacilli* dominant clusters (cluster 3 and 4) than those with persistent infection (29%), but the difference was not statistically significant (*p* value = 0.10) (Fig. 1b, Table 2).

**Table 2:** I. Distribution of HPV negative women, women with transient HPV16 infection and women with persistent HPV16 infection and CIN2+ in the four clusters. Proportion of total number of samples given in percent are reported within parenthesis. II. Statistical comparisons of the frequencies of non-Lactobacillus clusters (cluster 3 and 4) based on baseline samples of HPV16 negative women versus those with transient and persistent HPV16 infection, and for the follow up sample between women with transient HPV16 and persistent HPV16 infection

I. Distribution of samples per cluster				
	Cluster #1	Cluster #2	Cluster #3	Cluster #4
HPV negative	14 (43.8)	9 (28.1)	7 (21.9)	2 (6.3)
HPV16 transient, baseline sample	3 (11.5)	10 (38.5)	12 (46.2)	1 (3.8)
HPV16 persistent, baseline sample	13 (34.1)	11 (28.9)	11 (28.9)	3 (7.9)
HPV16 transient, follow up sample	4 (15.4)	11 (42.3)	10 (38.5)	1 (3.8)
HPV16 persistent, follow up sample	15 (39.5)	12 (31.6)	7 (18.4)	4 (10.5)
II. Statistical comparisons				
	*p* value	*q* value		
a. Baseline sample				
HPV16 negative versus transient and persistent HPV16 infection^a^	0.0173	0.0519		
Transient HPV16 versus persistent HPV16 infection^a^	0.1433	0.4299		
b. Follow up sample				
Transient HPV16 versus persistent HPV16 infection^a^	0.423	1		

*p* value for binomial testing

^a^Two-sided binomial testing

### Comparison of alpha diversity between infection groups

Alpha diversity was calculated for each sample using the Shannon index, as an indicator of the variability of the vaginal microbiota. There was no significant difference between HPV negative women, women with transient HPV16 infection, and those with persistent HPV16 infection, neither for the baseline sample (all three groups of women) nor the second sample (the second two groups). All nominal *p* values > 0.2.

### Transition rate between microbial profiles in women with transient or persistent HPV16 infection

**Fig. 2:**
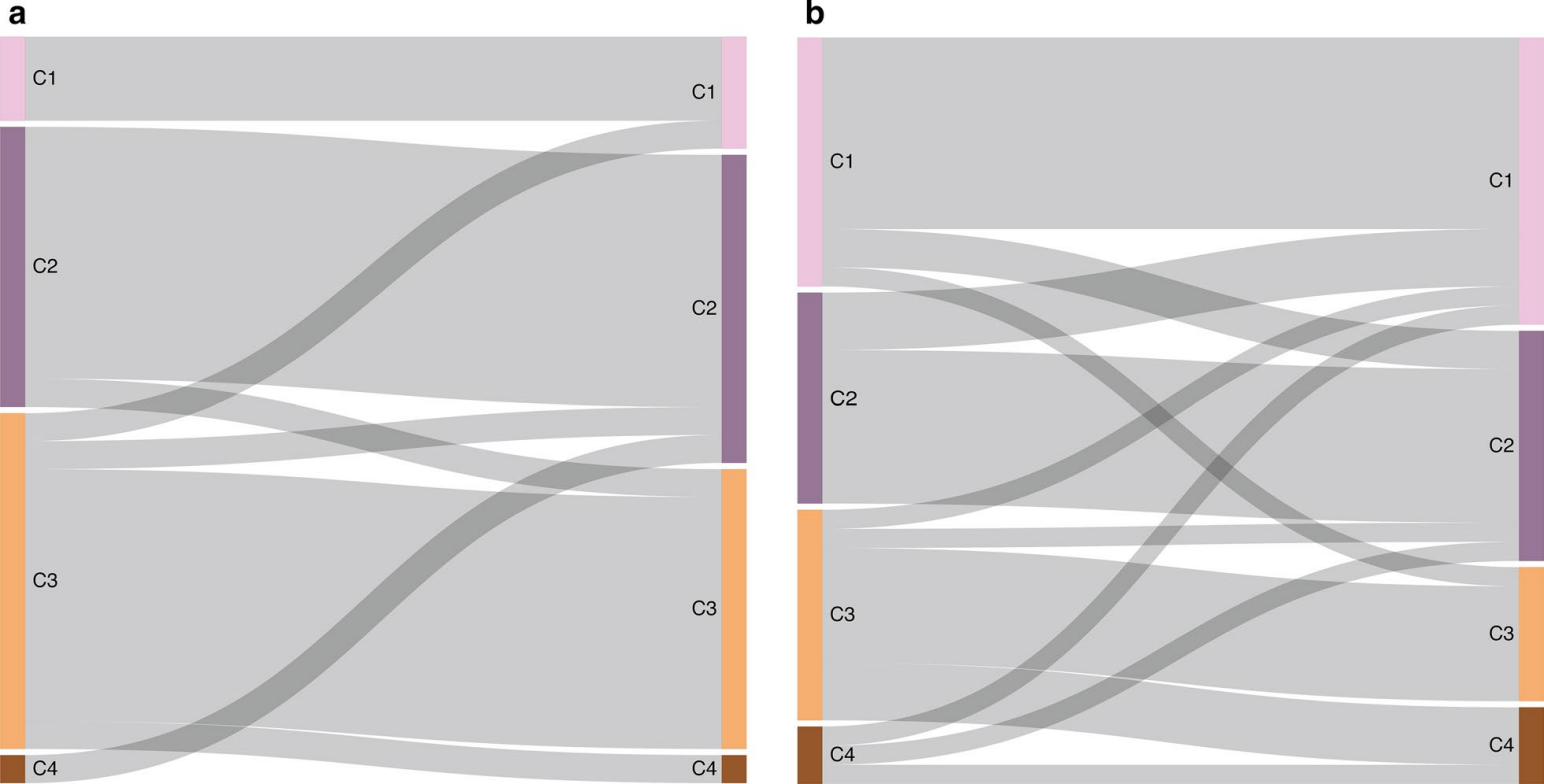
Sankey plot for transition events between baseline and follow up sample for K means clusters. Left column represents baseline sample and right column follow up sample. Color and text indicate K means cluster. **a** Women with HPV16 transient infection. **b** Women with persistent HPV16 infection and CIN2+

We compared the K-means clusters in the baseline and second samples of individual women to study the transition between clusters as a function of clearance and persistence of HPV16 infection. Transition between clusters were significantly more frequent in women with persistent HPV16 infection (34%) as compared to women who cleared the HPV16 infection in the timespan between the two sampling occasions (19%) (*p* value = 0.036, Fig. 2a,b).

## Discussion

A vaginal microbiota dominated by non-*Lactobacillus* species has been reported to be associated both with the risk of HPV infection and persistence of HPV infection [11–13] and is associated with up to three times higher odds for HPV infection and dysplasia [31]. In this study, we evaluated differences in vaginal microbiota in women with persistent single HPV16 infection, represented by women with two consecutive HPV16 positive tests and histologically diagnosed CIN2+ lesions, with women who cleared their HPV16 infection within 5–6 months, and a group of women who were HPV negative in the baseline test.

K-means clustering resulted in one cluster with *Lactobacillus sp.* dominance, a second cluster with *Lactobacillus Iners* dominance, a third cluster with a more diverse distribution of bacteria, and a fourth cluster also with a diverse distribution of bacteria but a higher abundance of *Gardnerella*. These groups are similar to previously described clusters [5, 6].

The results indicate that transient and persistent HPV16 infections, as compared to being HPV negative, is associated with a vaginal microbiota dominated by non-*Lactobacillus* species (*p* value = 0.0173). This is consistent with the results of several other studies [11–13]. However, the association did not remain significant after Bonferroni correction for multiple testing (*q* value = 0.0519). We did not detect a difference in alpha diversity between women with or without HPV16 infection, nor did we find a difference between women who cleared their HPV16 infection and those with persistent HPV16 infections. This is consistent with one previous study [32], but other studies have reported the opposite [33, 34].

The vaginal microbiota profile did not differ significantly when comparing women who cleared their infection with those that had a persistent infection and CIN2+. However, there was a significantly higher rate of transitioning between microbiota profiles in women with persistent HPV16 infection, as compared to women who cleared their infection. This indicate an instability in the vaginal microenvironment in women with a persistent HPV infection and CIN2+.

The causative relationship between HPV infection and vaginal microbiota is poorly understood, but the inflammatory process in women with CIN2+ could have an effect on the vaginal microenvironment and the composition of the vaginal microbiota. The initial HPV infection in the basal layer has no productive life cycle before the differentiation of the infected cells, it contains no virus induced cell-death and causes limited inflammatory response [35]. The HPV virus also have immune evasion mechanism. For example, it has been shown that HPV infection halt downstream release of signals from viral RNA receptors, resulting in an inhibition of the inflammatory process [36] and downregulates IL-1β, which is involved in the adaptive immune system [36, 37]. However, during a persistent HPV infection and development of CIN, the inflammatory process intensifies [38, 39], which could possibly influence the stability of the vaginal microenvironment and microbiota.

The strength of our study is that the women included were single-infected with HPV16, which reduces the complexity of HPV infection with different HPV types. We used HPV16 infected women that developed CIN2+ as a proxy for persistent infection. These women are likely to have harbored their HPV16 infections for some time, since they had developed CIN2+. There are some limitations in this study. The limited sample size reduces the statistical power to detect actual associations between the vaginal microbiota and the presence of HPV16. The HPV test include 12 high-risk HPV types, but the samples could possibly have other HPV infections that could affect the microbiota, which would go undetected with the test used in this study. Self-sampling could also potentially detect HPV-infections not exclusively present in the endocervix which would lead to an overestimation of detected HPV-infections. Here, this does not affect the negative or persistent group with CIN2+ histology diagnosis but may have influenced the positive HPV16 test for the first test in the transient group. A recent meta-review [40] does however conclude that PCR-based hrHPV tests on self-samples have similar sensitivity as clinician samples although a 2% lower specificity for CIN2+ could be observed.

We also lack information on whether the women participating in the study have been vaccinated for HPV or not. However, the age groups participating in the study have not been included in the organized Swedish HPV-vaccination program which was initiated in 2012 for girls born in 1998–2000 [41] and vaccination rates among the women participating in the current study, all born before 1986, are assumed to be low. Finally, we lack information about co-variates that could affect the vaginal microbiota, such as smoking status, menstrual cycle time point, sexual behavior, use of hormonal contraceptives or copper intrauterine devices and ethnicity [6, 16–19]. Previous investigations have however shown small, or no significant effects of the menstrual cycle stage or of use of hormonal contraceptives on the vaginal microbiota [42, 43] although effects have been observed coupled to the use of copper intrauterine devices [43]. Sexual behavior has also been shown to affect the vaginal microbiota [44] and not being able to adjust for these factors may also have reduced the statistical power to detect differences between study groups.

We used the seven variable regions included in the Ion 16S™ Metagenomics Kit. As noted, there is a difference in the number of OTUs identified at all taxonomic levels when the analysis is based on all seven variable regions as compared to the frequently employed regions V3 and V4. This can be attributed to the variability of the 16S gene, the differences in primer design and sequencing technology makes direct comparisons between studies of the vaginal microbiota difficult. However, the use of seven regions resulted in a higher number of OTUs identified and generally had a good correspondence with the CSTs previously described for the vaginal microbiota.

In summary, to our knowledge this is the first report showing a significantly higher rate of transitioning between vaginal microbiota profiles in women with persistent HPV16 infection and CIN2+ as compared to women who cleared their infection.

## Conclusion

Women with persistent HPV16 infection and CIN2+ have a higher rate (*p* value = 0.036) of transitions between microbial profiles than women with transient HPV16 infection. This indicate a higher instability in the vaginal microenvironment in women with persistent HPV infection and development of CIN2+.

## Supplementary information

**Additional file 1**. **Figure S1**: Venn diagram showing the overlap between the number of OTUs detected using seven variable regions (V2, V3, V4, V6, V7, V8 and V9) (blue) and the regions V3 and V4 only (yellow) based on the 16S rRNA Ion Torrent amplicon sequencing kit. Figures represents the taxonomic level: A. Species, B. Genus, C. Family.

## Data Availability

The 16S sequencing data (sequencing reads or microbiome profile) will be made available from the corresponding author (to U.G.) upon request.

## Abbreviations

CIN2+: Cervical intraepithelial neoplasia grade 2 or worse
CST: Community state types
HPV: Human papilloma virus
OTU: Operational taxonomic units
PCR: Polymerase chain reaction
