# Supplementary figures and images for: Temporal changes in the vaginal microbiota in self-samples and its association with persistent HPV16 infection and CIN2+
Source: Virol J. 2020 Oct 7;17:147. doi: 10.1186/s12985-020-01420-z (PMC7541248; doi:10.1186/s12985-020-01420-z)

## Additional file 1: Figures

Additional file 1: Figure S1.

**
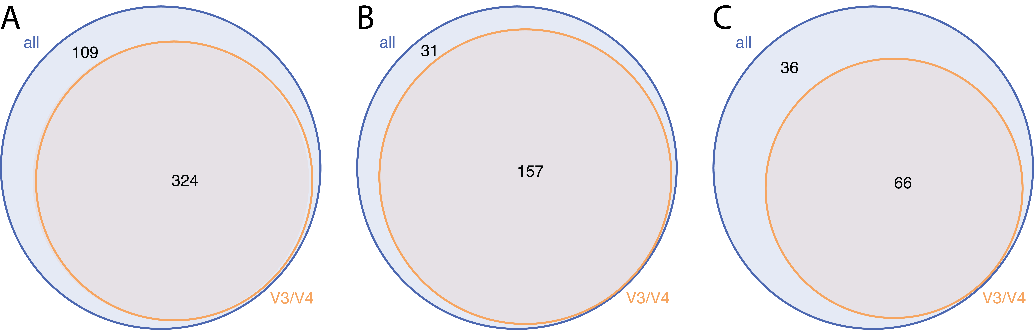
**

Supplement: Supplementary file 1 — Additional file 1. Figure S1: Venn diagram showing the overlap between the number of OTUs detected using seven variable regions (V2, V3, V4, V6, V7, V8 and V9) (blue) and the regions V3 and V4 only (yellow) based on the 16S rRNA Ion Torrent amplicon sequencing kit. Figures represents the taxonomic level: A. Species, B. Genus, C. Family. [file 12985_2020_1420_MOESM1_ESM.docx]
